# Application of cerebellar vermis theta-burst stimulation patterned repetitive transcranial magnetic stimulation in patients with chronic schizophrenia

**DOI:** 10.3389/fpsyt.2025.1729585

**Published:** 2026-01-12

**Authors:** Sihong Lei, Qiunan Lei, Jiumei Yang, Huaiping Peng, Jun Feng

**Affiliations:** 1 Department of Clinical Psychology, Beijing Anzhen Nanchong Hospital of Capital Medical University & Nanchong Central Hospital, Nanchong, China; 2 Department of Pain and Sleep Medicine, Nanchong Mental and Physical Health Hospital, Nanchong, China; 3 Department of Otorhinolaryngology, Beijing Anzhen Nanchong Hospital of Capital Medical University & Nanchong Central Hospital, Nanchong, China; 4 Department of Brain Functional Rehabilitation and Imaging, Beijing Anzhen Nanchong Hospital of Capital Medical University & Nanchong Central Hospital, Nanchong, China

**Keywords:** cerebellar vermis, chronic schizophrenia, cognitive function, repetitive transcranial magnetic stimulation, theta-burst stimulation

## Abstract

**Objective:**

To observe the therapeutic effects of cerebellar vermis theta-burst stimulation (TBS)-patterned repetitive transcranial magnetic stimulation (rTMS) in patients with chronic schizophrenia (CSZ).

**Methods:**

A total of 114 CSZ patients admitted to the hospital from February 2024 to July 2025 were enrolled and randomly divided into two groups (n=57 each). Both groups received conventional antipsychotic treatment, while the interventional group additionally underwent cerebellar vermis TBS-patterned rTMS, and the control group received sham stimulation. The treatment lasted for 4 weeks. The Positive and Negative Syndrome Scale (PANSS) score, Personal and Social Performance Scale (PSP) score, electrophysiological indexes of brain, MATRICS Consensus Cognitive Battery (MCCB) test and adverse reactions were compared between the two groups.

**Results:**

Following the intervention, both groups exhibited significant reductions in negative symptoms, general symptoms, and total PANSS scores, with the intervention group demonstrating significantly lower scores than the control group (*P* < 0.05). Similarly, Brief Psychiatric Rating Scale (BPRS) scores decreased and PSP scores increased in both groups, again with more favorable outcomes in the intervention group (*P* < 0.05). Neuroelectrophysiological measures (N2-P3 latency, N2-P3 amplitude, P300 latency and amplitude) and cognitive domains (information processing speed, working memory, attention/vigilance, and social cognition) improved in both groups, with significantly greater improvements observed in the intervention group compared to controls (*P* < 0.05). The incidence of adverse reactions did not differ significantly between groups (*P*>0.05).

**Conclusion:**

Cerebellar vermis TBS-patterned rTMS can effectively stimulate neural activity in CSZ patients, alleviate negative and general symptoms, improve disease control, enhance cognitive and social functioning, and demonstrate high safety.

## Introduction

Chronic schizophrenia (CSZ) is a clinically prevalent and severe mental disorder characterized by incoordination of mental activities, accompanied by impairments in cognition, thinking, and affect. It follows a chronic progressive course, and without timely and effective intervention, prolonged illness progression may lead to mental and cognitive decline, resulting in psychiatric disability. In severe cases, patients may lose the ability for self-care, making CSZ one of the top ten disabling diseases in China, imposing a substantial burden on both affected families and society (1). Currently, the primary clinical approach for CSZ involves long-term antipsychotic medication. While this can alleviate some clinical symptoms, the overall therapeutic efficacy remains suboptimal. Moreover, prolonged use of antipsychotics increases the risk of adverse effects, compromises medication adherence, and ultimately hinders effective disease management (2). Therefore, it is still necessary to seek safer and more effective treatment methods to enhance efficiency, better improve patients’ clinical symptoms, and restore normal social functions.

In recent years, with continued clinical research on CSZ treatment, non-pharmacological interventions have been widely adopted in clinical practice. Among these, repetitive transcranial magnetic stimulation (rTMS) has emerged as a prominent physical therapy modality. As a non-invasive neuromodulation technique, rTMS delivers targeted magnetic stimulation to specific brain regions, enhancing neuronal activity and metabolism while inducing cortical neuroplasticity. This mechanism effectively alleviates neuropsychiatric symptoms and has demonstrated significant therapeutic efficacy as an adjunctive treatment for mental disorders including depression and CSZ (3). And many previous studies have pointed out that rTMS can improve the negative symptoms of CSZ patients, and pointed out that different stimulation sites, stimulation modes and parameters will have a certain impact on the treatment effect (4). Theta-burst stimulation (TBS), a specific paradigm of rTMS, demonstrates superior efficacy in modulating synaptic plasticity and enhancing cortical excitability (5). Literature reported that human advanced functions such as emotions and cognition are closely related to the cerebellum (6). In patients with CSZ, multiple gray matter defects are often found in the cerebellar lobules, and there are abnormal connections in subcortical structures such as the thalamus and prefrontal lobe. The vermis of the cerebellum is an important part of the cerebellum, which is related to emotion and emotional regulation, and atrophy of the vermis can induce varying degrees of cognitive dysfunction (7). Therefore, more and more medical researchers in clinical practice have taken the vermis of the cerebellum as a new target for stimulation treatment of CSZ.

Positive and Negative Syndrome Scale (PANSS), Brief Psychiatric Rating Scale (BPRS), and MATRICS Consensus Cognitive Battery (MCCB) are widely used concise assessment tools in psychiatry, effectively reflecting the severity of patients’ conditions and cognitive function (8). However, the evaluation process of these scales involves a certain degree of subjectivity. In contrast, event-related potential (ERP) components such as N2-P3 and P300 serve as objective biological markers reflecting cognitive processing and information processing in the brain. Studies have confirmed that patients with CSZ exhibit significant reductions in P300 amplitude, prolongation of P300 latency, and abnormal changes in the N2-P3 component (9). These abnormalities are closely associated with the degree of cognitive impairment and can serve as noninvasive, objective indicators for assessing neural functional recovery. This study further observed the application effect of rTMS with theta burst stimulation (TBS) mode on the vermis of the cerebellum in patients with CSZ.

## Materials and methods

### Study subjects

A total of 114 CSZ patients admitted to our hospital between February 2024 and July 2025 were enrolled as study subjects and randomly assigned to two groups (n=57 each). Written informed consent was obtained from all participants. The study was approved by the Committees for the Ethical Review of Research involving Human Subjects from Nanchong Central Hospital (Approval No. NCCH-0016). This randomized controlled trial was prospectively registered at Chinese Clinical Trial Registry (identifier: ChiCTR2400081928).

### Inclusion and exclusion criteria

Inclusion criteria: (1) Fulfillment of the diagnostic criteria for CSZ (10); (2) Maintenance on a stable dosage of antipsychotic medication for at least three months; (3) BPRS score < 35 score (11) for over one year; (4) Completion of at least primary education, with the ability to read independently; (5) Undergoing rTMS treatment for the first time; (6) Demonstrated good compliance and capacity to cooperate with all study procedures; (7) Provision of written informed consent by both patients and their families.

Exclusion criteria: (1) Presence of other severe organic diseases; (2) Contraindications to rTMS treatment, including cochlear implants, cardiac pacemakers, or metallic implants within the body; (3) History of epilepsy; (4) Diagnosis of malignant tumors; (5) Receipt of electroconvulsive therapy within the 4 weeks prior to enrollment; (6) Comorbid other major psychiatric disorders; (7) Known intolerance to any medication used in this study. Participants who develop intolerance after randomization will have the medication discontinued and will be withdrawn from the study. Their data will be analyzed following the intention−to−treat (ITT) principle.

Participant Discontinuation: Participants could be discontinued from the treatment protocol for the following reasons: (1) Schizophrenia relapse during treatment; (2) Poor compliance and inability to complete the treatment; (3) Withdrawal from the study due to unforeseen circumstances. All randomized participants, including those who were discontinued from the treatment protocol, were retained in their originally assigned groups for the primary ITT analysis.

### Intervention methods

Both groups of patients received conventional antipsychotic drug treatment. They were orally administered Risperidone (Zhejiang Huahai Pharmaceutical Co., Ltd., NMPA Approval Number H20052330, specification: 1mg), with an initial dose of 1mg per time, twice a day, and the dosage was gradually increased to 3mg per time, twice a day; or Olanzapine (Qilu Pharmaceutical Co., LTD., National Drug Approval No. H20163165,specification: 10mg), with an initial dose of 10mg per time, once a day, and the dosage was gradually increased to 5-20mg per time.

The interventional group received rTMS treatment with the theta burst stimulation (TBS) mode on the vermis of the cerebellum. The rTMS treatment was conducted using a transcranial magnetic stimulation device (Manufacturer: TonicaElektronik A/S, Denmark; Model: Magpro R30, National Medical Device Injection 20162261853). An 8-coil stimulation head was used, and it was positioned 1 cm below the occipital protuberance of the patient (the vermis of the cerebellum). 100% of the motor threshold was used as the stimulation level. The TBS mode parameters were as follows: the basic frequency was 5 Hz, with one short burst stimulation every 200 ms, and each short burst contained three single pulses at a frequency of 50 Hz. There was an 8-second interval between every 10 short burst stimulations, and a total of 200 short burst stimulations were administered. The total number of stimulation pulses per day should be 600, with each treatment lasting 200 seconds. The treatment was administered once a day, 5 days a week, for a continuous period of 4 weeks.

The control group received sham stimulation treatment. The stimulation magnetic head was reversed to maintain a 180° angle with the scalp for stimulation, while other related stimulation parameters remained unchanged. The treatment was conducted continuously for 4 weeks.

To assess blinding integrity, participants were asked to guess their group assignment (real or sham) at the end of the treatment. The accuracy of guesses was analyzed using chi-square test.

### Observation indicators

1. Mental symptoms: Before and after treatment, PANSS (12) was used to evaluate, including positive symptoms, negative symptoms and general symptoms, with 7, 7 and 16 items, 49, 49 and 112 points respectively, with 1-7 points for each item and the highest score of 210 points. Mental symptoms were positively correlated with the score.
2. Disease assessment: Before and after treatment, the BPRS was used for assessment, consisting of 18 items, each scored on a 7-point scale (1-7), with a total score of 126 points. The higher the score, the more severe the condition.
3. Social function: Before and after treatment, the Personal and Social Performance Scale (PSP) (13) was used for assessment, covering 4 dimensions: self-care, useful activities in society, disruptive and aggressive behaviors, and personal and social relationships, with a total score of 100 points. The higher the score, the more severe the social function impairment.
4. Neuroelectrophysiological indicators: Before and after treatment, the N2-P3 and P300 latency and amplitude of patients were detected using the Bravo brain evoked potential instrument.
5. Cognitive function: Before and after treatment, the MCCB (14) was used for assessment, which contains 7 factors: visual learning, information processing speed, working memory, verbal learning, attention/vigilance, social cognition, reasoning and problem-solving. The better the cognitive function, the higher the score. Among them, the Continuous Performance Test needs to be completed on a computer under the unified guidance of the investigator, while other tests are evaluated by professional investigators.
6. Adverse Effects: Monitored effects included dizziness, insomnia, headache, etc.

### Statistical analysis

Statistical analyses were performed using SPSS 25.0 software. All analyses were conducted based on the ITT principle, with the last observation carried forward (LOCF) method used to handle missing data for participants who withdrew. Categorical data were expressed as frequency (n) and percentage (%) and compared between groups using the Chi-square test. Continuous data were expressed as mean ± standard deviation. To compare the changes in outcomes between the two groups over time, repeated-measures analysis of variance (RM-ANOVA) was employed, with time (pre-treatment, post-treatment) as the within-subject factor and group (intervention, control) as the between-subject factor. The group × time interaction effect was examined to determine if the change over time differed between groups. If a significant interaction was found, simple effects analyses were performed to compare groups at each time point and to compare time points within each group. For baseline comparisons between groups, independent samples t-tests were used for continuous variables and chi-square tests for categorical variables. Additionally, an analysis of covariance (ANCOVA) was performed on post-treatment scores, with baseline scores and chlorpromazine equivalent dose as covariates, to control for potential confounding effects of medication dosage. A two-sided *P*-value < 0.05 was considered statistically significant.

## Results

### Baseline characteristics

A total of 114 patients were randomized. The accuracy of participant guesses regarding group assignment did not differ significantly from chance (Intervention group: 52.6% correct guess; Control group: 49.1% correct guess; χ² = 0.145, P = 0.703), suggesting that blinding was successful.

All randomized participants were included in the ITT analysis. Five participants (2 in the intervention group, 3 in the control group) withdrew during the study (1 due to relapse, 2 due to non-compliance, 2 due to personal reasons). Their last available data were carried forward. Baseline characteristics were well-balanced between interventional group and control group (all *P* > 0.05, **Table 1**).

**Table 1:** Baseline characteristics of between interventional group and control group.

Characteristic	Intervention group (n=57)	Control group (n=57)	*t*/*χ*^2^	*P*
Age (years)	43.65 ± 5.82	44.59 ± 5.79	0.865	0.389
Gender, n (%)			0.336	0.562
Male	20 (35.09)	23 (40.35)		
Female	37 (64.91)	34 (59.65)		
Disease duration (years)	6.24 ± 0.68	6.29 ± 0.71	0.384	0.702
Annual relapse frequency	3.25 ± 0.21	3.18 ± 0.23	1.697	0.093
Chlorpromazine equivalent dose (mg)	472.58 ± 58.46	468.62 ± 58.37	0.362	0.718
Education level, n (%)			0.144	0.704
Junior high school or below	34 (59.65)	32 (56.14)		
Senior high school or above	23 (40.35)	25 (53.86)		
Family history, n (%)			0.134	0.714
Positive	3 (5.26)	5 (8.77)		
Negative	54 (94.74)	52(91.23)		

### PANSS score

The RM-ANOVA revealed a significant group × time interaction effect for negative symptoms (*P* < 0.001), general symptoms (*P* < 0.001), and total PANSS score (*P* < 0.001). Following therapy, the scores of two groups for negative symptoms, and general symptoms, as well as the total score all decreased. Simple effects analysis showed that at post-treatment, the intervention group had significantly lower scores than the control group for negative symptoms (*P* < 0.001), general symptoms (*P* < 0.001), and total score (*P* < 0.001). There was no significant interaction for positive symptoms (*P* = 0.910) (**Table 2**).

**Table 2:** Comparison of PANSS score between the two groups before and after treatment ( ± *s*, score).

Group	Time point	Positive symptoms	Negative symptoms	General symptoms	Total score
Control group (n=57)	Before treatment	14.85 ± 1.25	22.46 ± 2.34	34.65 ± 3.42	71.98 ± 5.34
	After treatment	14.25 ± 1.17	15.67 ± 1.67^a^	30.68 ± 2.71^a^	60.59 ± 4.37^a^
Interventional group(n=57)	Before treatment	14.79 ± 1.22	22.51 ± 2.37	34.58 ± 3.39	71.88 ± 5.31
	After treatment	14.18 ± 1.14	12.75 ± 1.34^a^	28.76 ± 1.85^a^	55.69 ± 3.42^a^
RM-ANOVA Group×Time Interaction (P-value)		0.910	<0.001	<0.001	<0.001
Between-group comparison at after treatment (P-value)		0.747	<0.001	<0.001	<0.001

PANSS, Positive and Negative Symptom Scale; The same group was compared before and after treatment: ^a^*P* < 0.05.

### BPRS and SANS score

A significant group × time interaction was found for both BPRS (*P* < 0.001) and PSP scores (*P* < 0.001). Following treatment, BPRS scores decreased and PSP scores increased in both groups. Simple effects analysis indicated that at post-treatment, the intervention group had significantly lower BPRS scores (*P* < 0.001) and higher PSP scores (*P* < 0.001) than the control group (**Table 3**).

**Table 3:** Comparison of BPRS and SANS scores between the two groups before and after treatment ( ± *s*, score).

Group	Time point	Positive symptoms	Negative symptoms
Control group (n=57)	Before treatment	33.24 ± 1.65	42.61 ± 4.62
	After treatment	31.06 ± 1.24^a^	54.64 ± 5.52^a^
Interventional group(n=57)	Before treatment	33.37 ± 1.68	42.64 ± 4.65
	After treatment	29.76 ± 0.86^a^	60.42 ± 6.34^a^
RM-ANOVA Group×Time Interaction (P-value)		<0.001	<0.001
Between-group comparison at after treatment (P-value)		<0.001	<0.001

BPRS score, Brief Mental Rating Scale (BPRS); PSP score, personal and social function scale; The same group was compared before and after treatment: ^a^*P* < 0.05.

### Brain electrophysiological index

Significant group×time interactions were observed for N2-P3 latency (*P* < 0.001), N2-P3 amplitude (*P* = 0.001), P300 latency (*P* < 0.001), and P300 amplitude (P<0.001). Following treatment, there was an increase in N2-P3 latency, N2-P3 amplitude, and P300 amplitude and a decrease in P300 latency in both groups. Simple effects analysis showed that at post-treatment, the intervention group had significantly higher N2-P3 latency (*P* < 0.001), N2-P3 amplitude (*P* < 0.001), and P300 amplitude (*P* < 0.001), and lower P300 latency (*P* < 0.001) compared to the control group (**Table 4**).

**Table 4:** Comparison of PSP scores between the two groups before and after treatment ( ± *s*, score).

Group	Time point	N2-P3 latency (ms)	N2-P3 amplitude (μV)	P300 latency (ms)	P300 amplitude (μV)
Control group (n=57)	Before treatment	75.12 ± 4.35	6.86 ± 0.57	337.46 ± 19.25	6.34 ± 0.42
	After treatment	80.34 ± 5.43^a^	8.11 ± 0.65^a^	324.46 ± 15.43^a^	7.65 ± 0.57^a^
Interventional group(n=57)	Before treatment	74.98 ± 4.31	6.82 ± 0.54	338.21 ± 19.30	6.30 ± 0.39
	After treatment	87.28 ± 6.43^a^	8.57 ± 0.74^a^	307.45 ± 12.37^a^	8.74 ± 0.65^a^
RM-ANOVA Group×Time Interaction (P-value)		<0.001	<0.001	<0.001	<0.001
Between-group comparison at after treatment (P-value)		<0.001	<0.001	<0.001	<0.001

The same group was compared before and after treatment: ^a^*P* < 0.05.

### Cognitive function

Significant group×time interactions were found for information processing speed (*P* < 0.001), working memory (*P* = 0.032), attention/vigilance (*P* < 0.001), and social cognition (*P* < 0.001). After treatment, these scores improved in both groups. Simple effects analysis revealed that at post-treatment, the intervention group scored significantly higher than the control group in information processing speed (*P* < 0.001), working memory (*P* = 0.032), attention/vigilance (*P* < 0.001), and social cognition (*P* < 0.001). No significant interactions were observed for visual learning, verbal learning, or reasoning and problem solving (all *P*>0.05) (**Table 5**).

**Table 5:** Comparison of MCCB score between the two groups before and after treatment ( ± *s*, score).

Group	Time point	Visual learning	Information processing speed	Working memory	Word learning	Attention/alertness	Social cognition	Reasoning and problem solving
Control group (n=57)	Before treatment	51.76 ± 4.28	34.62 ± 3.65	39.86 ± 2.64	42.16 ± 3.24	37.65 ± 1.34	39.34 ± 3.11	46.74 ± 4.36
	After treatment	50.75 ± 3.86	36.75 ± 2.43^a^	42.67 ± 3.42^a^	43.68 ± 4.15	41.58 ± 2.14^a^	42.68 ± 4.15^a^	47.29 ± 5.37
Interventional group(n=57)	Before treatment	51.58 ± 4.24	34.58 ± 3.62	39.83 ± 2.61	42.18 ± 3.28	37.58 ± 1.30	39.42 ± 3.15	46.72 ± 4.32
	After treatment	49.62 ± 3.86	38.46 ± 1.85a^a^	44.27 ± 4.37^a^	44.15 ± 4.20	45.75 ± 3.25^a^	47.58 ± 5.28^a^	48.35 ± 5.42
RM-ANOVA Group×Time Interaction (P-value)		0.822	<0.001	0.032	0.974	<0.001	<0.001	0.980
Between-group comparison at after treatment (P-value)		0.121	<0.001	0.032	0.549	<0.001	<0.001	0.297

MCCB score, MATRICS Consensus Cognitive Battery (MCCB); The same group was compared before and after treatment: ^a^*P* < 0.05.

### Adverse effect

Adverse reactions occurred in 4 patients (7.02%) in the control group and 5 patients (7.02%) in the intervention group, with no statistically significant difference in overall incidence between the two groups (*P*>0.05) (**Table 6**).

**Table 6:** Comparison of the incidence of adverse reactions between the two groups n(%).

Group	Dizzy	Insomnia	Headache	Blood freezes	Total occurrence
Control group (n=57)	1(1.75)	1(1.75)	1(1.75)	1(1.75)	4(7.02)
Interventional group(n=57)	2(3.51)	1(1.75)	1(1.75)	1(1.75)	5(7.02)
*χ^2^*					0.000
*P*					1.000

## Discussion

Currently, the precise pathogenesis and etiology of CSZ remain unclear, resulting in significant therapeutic challenges and the absence of definitive curative treatments. Clinical management primarily relies on antipsychotic medications for symptom control. However, long-term pharmacotherapy presents difficulties in dosage optimization and is frequently associated with adverse drug reactions, which may compromise treatment adherence. Notably, a substantial proportion of patients continue to exhibit persistent psychiatric symptoms post-treatment, particularly prominent negative symptoms and cognitive impairments that severely impair social functioning and reduce psychosocial competence (15, 16). Therefore, further exploring efficient and safe non-drug treatment methods has become a hot spot in clinical neurology.

RTMS is a non-drug physical intervention method, which can give magnetic stimulation to specific parts of the brain to promote the remodeling of cerebral cortex function and improve neurological symptoms. It has been widely used in the treatment of many mental diseases in clinic and achieved good results (17). There are various rTMS modes, while TBS is a new type of rTMS stimulation mode, which can induce the long-term enhancement effect of synaptic transmission in central nervous system and enhance the excitability of cerebral cortex by simulating the spike release mode of hippocampal neurons in animal inquiry behavior. Compared with the traditional rTMS mode, it has lower stimulation intensity and shorter single treatment time, which can induce the excitability of cerebral cortex to be prolonged, so as to obtain better stimulation treatment effect (18). Neuroelectrophysiological abnormalities often occur in the brain of CSZ patients, and P300, N2-P3 latency and amplitude play an important role in the assessment of EEG abnormalities, among which N2-P3 is an important electrophysiological index in the process of gaining confidence and cognition. If N2-P3 latency and amplitude are prolonged, it suggests that the process of gaining confidence and cognition is improved, while the change of P300 can reflect the neurophysiological changes in the process of working memory and psychological processing when the brain performs cognitive tasks (19, 20).This study found that after treatment, the negative symptoms, general symptoms, and BPRS scores of the interventional group were lower than those of the control group. The N2-P3 latency, N2-P3 amplitude, and P300 amplitude of the interventional group were higher than those of the control group, while the P300 latency was lower. This suggests that rTMS treatment with the TBS mode on the vermis of the cerebellum can regulate the physiological brain waves of patients with CSZ and improve negative symptoms and general symptoms. The reason for this analysis is that in this study, the rTMS stimulation of the cerebellar vermis was conducted using the TBS mode. This area is located in the central position of the cerebellum and can regulate the activity of the human brain’s limbic system, participating in the regulation of individual cognition and emotions. High-frequency magnetic stimulation of the cerebellar vermis can reduce the excitability of the dentate nucleus, activate the excitability of neurons between the cerebral cortex regions, and further activate the frontal lobe of the brain related to human emotions and cognition through the transmission of dopamine and serotonin between neurons. This, in turn, regulates the electrophysiological indicators of brain nerves, prolongs the amplitude of brain electrical physiological waves, and better promotes the improvement of negative symptoms in patients and controls the condition (21).

According to literature reports, approximately 85% or more of patients with schizophrenia exhibit cognitive impairments across multiple domains, including working memory and executive function (22). Furthermore, these cognitive deficits tend to progressively worsen with the chronic progression of the disease, significantly impacting patients’ daily functioning. This study evaluated the cognitive and social functions of patients with CSZ, the results showed that after treatment, the PSP score of the interventional group was higher than that of the control group, and the scores of information processing speed, working memory, attention/vigilance, and social cognition were also higher than those of the control group. This suggests that rTMS treatment with the TBS mode on the vermis of the cerebellum can improve the cognitive function of patients with CSZ and promote the recovery of social function. The reason for this is that the rTMS treatment with the TBS mode on the vermis of the cerebellum can plastically regulate the neural synapses of the vermis of the cerebellum and stimulate the prefrontal lobe of the brain. The prefrontal lobe is an important neurophysiological basis for emotional control, autonomic nervous system regulation, and social cognition in the human brain. Therefore, the rTMS treatment with the TBS mode can effectively regulate the emotional and cognitive states of CSZ patients, improve their brain information processing, working memory and cognitive functions, thereby enhancing their cognitive functions, enabling them to adapt to daily life and society as soon as possible, and promoting the recovery of social functions (23, 24). This result shows that there was no difference in adverse reactions between the two groups, suggesting that the rTMS treatment with the TBS mode on the vermis of the cerebellum for CSZ is highly safe. This might be due to the short treatment time of the TBS mode of rTMS, which can avoid the excessive cortical activity caused by long-term brain magnetic stimulation and reduce adverse conditions such as transdermal numbness, insomnia, and dizziness caused by magnetic stimulation.However, this study still has many deficiencies. For instance, the sample size included was small, and the cases were from a single source, which may lead to certain deviations in the research results. Moreover, this study only conducted short-term result analysis, and the long-term efficacy of rTMS treatment with the TBS mode targeting the vermis of the cerebellum for CSZ remains unknown. In the future, multi-center, large-sample studies are still needed, and the follow-up time after treatment should be extended to further analyze the long-term therapeutic effect in depth, so as to provide more objective guidance for the selection of treatment plans for CSZ.

In conclusion, the application of rTMS with TBS mode on the vermis of the cerebellum in patients with CSZ can effectively regulate their neurological functions, alleviate the condition, improve negative and general symptoms, promote the enhancement of cognitive and social functions of the patients, and has high treatment safety. It can be promoted and applied in clinical practice.

## Data Availability

The raw data supporting the conclusions of this article will be made available by the authors, without undue reservation.
